# Supplementary material for: WordCommentsAnalyzer: A windows software tool for qualitative research
Source: F1000Res. 2018 Sep 4;7:536. Originally published 2018 May 3. [Version 2] doi: 10.12688/f1000research.14819.2 (PMC6137411; doi:10.12688/f1000research.14819.2)
Supplement: Supplementary file 3 [file f1000research-7-17614-s0002.tgz › b2a79496-4ada-47c4-9682-a99ce867836d.zip!/Eight Iranian Health Journals/Salmand_Iranian Journal of Ageing _1.docx]

© Salmand_Iranian Journal of Ageing . This work is licensed under CC BY-NC 4.0 (https://creativecommons.org/licenses/by-nc/4.0/).

Volume & Issue: دوره 13 - شماره 1

Title: طراحی و اجرا و ارزیابی آموزش مبحث دندان‌پزشکی سالمندان به صورت مجازی به دانشجویان دندان‌پزشکی

http://salmandj.uswr.ac.ir/article-1-1227-fa.html

اهداف امروزه آموزش دندان‌پزشکی سالمندان به دانشجویان دندان‌پزشکی اهمیت یافته است. از سوی دیگر با مطرح‌شدن تکنولوژی‌های نوین آموزشی، تأکید بسیاری بر تغییر رویکرد از شیوه آموزش سنتی به شیوه مجازی شده است. این مطالعه در سال تحصیلی 1394-1395 با هدف طراحی و اجرا و ارزیابی آموزش مبحث دندان‌پزشکی سالمندان به صورت مجازی به دانشجویان سال آخر دندان‌پزشکی دانشگاه علوم پزشکی تهران و مقایسه آن با روش سنتی انجام شده است. مواد و روش ها در این مطالعه مداخله‌ای، 64 نفر از دانشجویان سال آخر دندان‌پزشکی شرکت کردند. مبحث دندان‌پزشکی سالمندان برای 31 نفر از دانشجویان شرکت‌کننده به شیوه مجازی و برای 33 نفر به شیوه سنتی ارائه شد. دانشجویان پرسش‌نامه‌ پیش‌آزمون را قبل از ارائه محتوای آموزشی تکمیل کردند. این پرسش‌نامه حاوی سؤالاتی در زمینه دانش و نگرش عمومی درباره دوران سالمندی و دانش و نگرش و عملکرد مرتبط با سلامت دهان سالمندان بود و روایی و پایایی آن قبلاً تأیید شده بود. پس از مداخله، دانشجویان پرسش‌نامه پس‌آزمون را تکمیل کردند. تجزیه‌وتحلیل آماری با استفاده از نرم‌افزار SPSS نسخه 21 و آزمون‌های کوواریانس و رگرسیون انجام شد. یافته ها میانگین نمرات، پس از مداخله گروه‌های آموزش سنتی و مجازی در حیطه‌های دانش و نگرش عمومی درباره دوران سالمندی و نیز دانش و نگرش مرتبط با سلامت دهان و دندان سالمندان، تفاوت معناداری با یکدیگر نداشت. در بخش عملکرد خوداظهار، نمرات دانشجویان گروه آموزش سنتی و مجازی قبل از مداخله به ترتیب 20+4/45 و 20+9/52 از 100 بود که بعد از مداخله به ترتیب به 26+9/56 و 21+4/75 افزایش یافت. در این بخش تفاوت معناداری بین دو گروه وجود داشت ( 05/0>P). نتیجه گیری شیوه آموزش مجازی به اندازه روش سنتی اهداف آموزشی را تأمین می‌کند. بنابراین می‌توان در تدریس مبحث دندان‌پزشکی سالمندان، آموزش مجازی را جایگزین آموزش سنتی کرد.

Title: تأثیر یک دوره تمرین مقاومتی بر فشار خون و نیتریک‌اکساید در زنان سالمند

http://salmandj.uswr.ac.ir/article-1-1166-fa.html

اهداف یکی از بیماری‌های شایع پس از یائسگی تصلب شرایین است. عملکرد اندوتلیال عامل مهمی در ارزیابی بیماری‌های تصلب شرایین و رسوب چربی در دیواره داخلی عروق و فشار خون و نارسایی قلبی است. سلول‌های اندوتلیال عروقی نقش مهمی در تنظیم فعالیت‌های عروقی با تولید مواد فعال‌کننده عروق، مانند نیتریک‌اکساید بر‌عهده دارند. مواد و روش ها این تحقیق تأثیر یک دوره تمرینات مقاومتی بر غلظت نیتریک‌اکساید و فشار خون زنان سالمند را بررسی می‌کند. 24 زن یائسه (با میانگین سنی 02/37±6/67، شاخص توده بدن 16/±4‌87/26 و نسبت کمر به باسن 4/0‌±‌92/0) به صورت هدفمند و تصادفی، انتخاب و به دو گروه 12نفره کنترل و تجربی تقسیم شدند. گروه تجربی در هشت هفته تمرین مقاومتی (هر هفته سه جلسه) با شدت 40 تا 65 درصد یک تکرار بیشینه شرکت کردند. قبل و بعد از برنامه تمرینی متغیرهای تحقیق اندازه‌گیری شد. از آزمون‌های آماری t همبسته مستقل برای ارزیابی یافته‌ها استفاده و سطح معنی‌داری کمتر از 05/0 در نظر گرفته شد. یافته ها نتایج این تحقیق نشان داد یک دوره تمرین مقاومتی تأثیر معناداری بر غلظت نیتریک‌اکساید پلاسمایی (01/0=P) و فشار خون سیستولی (006/0=P) و دیاستولی زنان سالمند دارد (002/0=P).  نتیجه گیری به نظر می‌رسد یک دوره تمرین مقاومتی با این شدت و حجم می‌تواند در کاهش فشار خون سیستولی و دیاستولی و نیز افزایش نیتریک‌اکساید پلاسما به عنوان یک عامل مهم برای جلوگیری از بیماری‌های قلبی‌عروقی و آترواسکلروز و پُرفشار خونی زنان سالمند مؤثر باشد.

Title: رابطه خودپنداره و خودکارآمدی با خودمدیریتی در سالمندان آسایشگاه‌های شهر تهران

http://salmandj.uswr.ac.ir/article-1-1124-fa.html

اهداف خودپنداره، خودکارآمدی و خودمدیریتی از مهم‌ترین متغیرهای حوزه سالمندی هستند که در آسایشگاه‌های سالمندی به طور ویژه به آن‌ها توجه می‌شود. هدف از این مطالعه تعیین رابطه خودپنداره و خودکارآمدی با خودمدیریتی سالمندان آسایشگاه‌های شهر تهران در سال 1394 بود. مواد و روش ها در این مطالعه توصیفی‌تحلیلی جامعه پژوهش شامل تمامی سالمندان مقیم آسایشگاه‌های شهر تهران در سال 1394بود. حجم نمونه با استفاده از فرمول کوکران 217 نفر محاسبه شد. روش نمونه‌گیری تصادفی ساده بود. برای جمع‌آوری داده‌ها از مقیاس خودپنداره راجرز، مقیاس خودکارآمدی عمومی و پرسش‌نامه خودمدیریتی (SMAS-30) استفاده شد. داده‌های جمع‌آوری‌شده وارد نرم‌افزار SPSS شد و با آزمون‌های کلموگروف اسمیرنوف، همبستگی پیرسون و رگرسیون تجزیه‌وتحلیل شدند. یافته ها در این پژوهش 217 سالمند (144 زن و 73 مرد) با میانگین سنی 78/11±66/78 سال به عنوان نمونه حضور داشتند. میانگین خودپنداره، خودکارآمدی و خودمدیریتی آنان به ترتیب 47/1±25/8، 00/4±68/17 و 59/11±69/59 بود. نتایج آزمون همبستگی نشان داد خودپنداره و خودکارآمدی با خودمدیریتی رابطه معنادار داشتند (05/0P<) و خودپنداره و خودکارآمدی حدود 14 درصد خودمدیریتی را تبیین می‌کردند. نتیجه گیری سالمندان مقیم آسایشگاه‌های شهر تهران خودپنداره ضعیف، خودکارآمدی کم و خودمدیریتی نامطلوبی داشتند. با توجه به روابط به‌دست‌آمده، به دست‌اندرکاران و مسئولان مربوطه پیشنهاد می‌شود برای افزایش خودمدیریتی سالمندان مقیم آسایشگاه‌های شهر تهران به خودپنداره، خودکارآمدی و راهکارهای ارتقای آن‌ها توجه بیشتری داشته باشند.

Title: نقش عملکرد در آزمون استروپ در پیش‌بینی اضطراب و خودکارآمدی در افتادن در سالمندان شهر اردبیل

http://salmandj.uswr.ac.ir/article-1-1419-fa.html

اهداف امروزه به دلیل افزایش امید به زندگی، به پدیده سالمندی بیش از پیش توجه شده است. پژوهش حاضر با هدف بررسی نقش عملکرد استروپ با اضطراب و خودکارآمدی در افتادن در سالمندان بیش از 60 سال انجام گرفت. مواد و روش ها طرح پژوهش توصیفی از نوع همبستگی بود. جامعه آماری شامل تمامی سالمندان مرد بیش از 60 سال ساکن در سرای سالمندان شهر اردبیل در سال 1396 بود که از بین آن‌ها نمونه‌ای به حجم 200 نفر به روش نمونه‌گیری دردسترس انتخاب شدند. برای جمع‌آوری اطلاعات از آزمون‌های اضطراب خودکارآمدی در افتادن و استروپ استفاده شد. داده‌ها با استفاده از آزمون همبستگی پیرسون و رگرسیون تحلیل شد.   یافته ها یافته‌های پژوهش نشان داد که رابطه معناداری بین عملکرد استروپ (کارت سوم و چهارم) در هر چهار خرده‌مقیاس (خطا، صحیح، واکنش، تداخل) با میزان اضطراب و خودکارآمدی در افتادن در سالمندان (01/0>P) وجود دارد. همچنین هر دو متغیر اضطراب و خودکارآمدی در افتادن قابل پیش‌بینی با عملکرد استروپ (01/0>P) است. نتیجه گیری با توجه به یافته‌های حاضر می‌توان نتیجه گرفت که با برگزاری کلاس‌های آموزشی و انجام گروه‌درمانی‌های مختلف می‌توان میزان عملکرد استروپ را در سالمندان بهبود بخشید تا با این کار به صورت غیرمستقیم میزان اضطراب را کاهش و خودکارآمدی در افتادن را در سالمندان افزایش داد.

Title: رابطه حمایت اجتماعی و جهت‌گیری مذهبی با رضایت از زندگی در سالمندان

http://salmandj.uswr.ac.ir/article-1-1189-fa.html

اهداف سالمندشدن جمعیت پدیده‌ای رو به افزایش است. در این سنین کاهش روابط اجتماعی بر نحوه زندگی و میزان رضایت از زندگی تأثیر می‌گذارد. پژوهش حاضر با هدف بررسی رابطه حمایت اجتماعی و جهت‌گیری مذهبی با رضایت از زندگی در سالمندان انجام شد. مواد و روش ها روش این مطالعه توصیفی از نوع همبستگی بود. جامعه آماری شامل تمامی سالمندان ساکن در خانه سالمندان شهرکرمانشاه در سال 1395 بود که 126 نفر (میانگین76/69 و انحراف استاندارد 9) به روش نمونه‌گیری در دسترس انتخاب شدند. ابزار جمع‌آوری شامل پرسش‌نامه‌های حمایت اجتماعی فیلیپس و همکاران (1986)، جهت‌گیری مذهبی آلپورت و راس (1967) و رضایت از زندگی داینر و همکاران (1985) بود. برای تحلیل داده‌ها از ضریب همبستگی پیرسون و تحلیل رگرسیون هم‌زمان و نرم‌افزارآماری SPSS استفاده شد. یافته ها نتایج بیانگر آن است که بین متغیر جهت‌گیری مذهبی با رضایت از زندگی در سطح 05/0P< رابطه وجود داشت و بین جهت‌گیری مذهبی بیرونی با رضایت از زندگی 077/0، جهت‌گیری درونی با رضایت از زندگی 249/0 در سطح 05/0P< همبستگی وجود داشت. میزان ارتباط جهت‌گیری درونی با حمایت اجتماعی 708/0 و جهت‌گیری بیرونی با حمایت اجتماعی 374/0 در سطح 05/0P< بود. بین حمایت اجتماعی و رضایت از زندگی رابطه وجود نداشت. جهت‌گیری مذهبی درونی، جهت‌گیری مذهبی بیرونی و حمایت اجتماعی قادر به پیش‌بینی واریانس رضایت از زندگی بود (R2=11%) نتیجه گیری با توجه به نیازی که سالمندان به حمایت اطرافیان دارند، با افزایش حمایت اجتماعی از این افراد و پایبندی آن‌ها به گرایش‌های مذهبی می‌توان برای ارتقای رضایت از زندگی آن‌ها گامی مؤثر برداشت.

Title: بررسی اثربخشی گروه‌درمانی شناختی وجودگرا بر امید به زندگی و افسردگی سالمندان مقیم مراکز سالمندی

http://salmandj.uswr.ac.ir/article-1-1269-fa.html

اهداف کاهش امید به زندگی و افسردگی از رایج‌ترین حالات روان‌شناختی در سالمندان است که می‌تواند واکنش های احتمالی متعددی به دنبال داشته باشد. هدف پژوهش حاضر بررسی تأثیر گروه‌درمانی با رویکرد شناختی وجودگرا بر کاهش میزان افسردگی و افزایش امید به زندگی در سالمندان است.  مواد و روش ها طرح این پژوهش طرح آزمایشی پیش‌آزمون و پس‌آزمون با گمارش تصادفی است. جامعه آماری این پژوهش شامل تمامی سالمندان مقیم مراکز شبانه‌روزی شهر کرمان بود. از این جامعه دو مرکز امیرالمؤمنین و مهرآذین به‌طور تصادفی انتخاب شد. سپس با مراجعه به این دو مرکز، 30 نفر (18 زن و 12 مرد) به طور تصادفی انتخاب و به دو گروه آزمایش و کنترل (هر کدام 15 نفر) تقسیم شدند. به گروه آزمایش درمان گروهی شناختی ‌وجودگرا در ده جلسه (هر جلسه 90 دقیقه) آموزش داده شد. گروه کنترل در این مدت مداخله‌ای دریافت نکردند. داده‌ها با استفاده از میانگین، انحراف استاندارد و تحلیل کواریانس تجزیه‌وتحلیل شد.  یافته ها نتایج نشان داد گروه‌درمانی شناختی وجودگرا بر امید به زندگی و افسردگی سالمندان مقیم مراکز سالمندی تأثیر دارد (P<0/01).  نتیجه گیری گروه‌درمانی شناختی وجودگرا با آموزش تصور از خویشتن، تأکید بر زمان حال و آگاهی‌دادن درباره مرگ به سالمندان، باعث افزایش امید به زندگی و کاهش میزان افسردگی و به دنبال آن افزایش سلامت روانی سالمندان می‌شود.

Title: تأثیر هشت هفته تمرین تناوبی هوازی بر سطح سرمی نیتریک اکساید و اندوتلین-1 مردان سالمند با اضافه وزن

http://salmandj.uswr.ac.ir/article-1-1323-fa.html

اهداف فعالیت‌های بدنی مختلف می‌تواند در بهبود سلامتی و ارتقای سطح آمادگی قلبی‌عروقی افراد نقش مهم و ارزنده‌ای داشته باشد. هدف از این پژوهش تأثیر هشت هفته تمرین تناوبی هوازی بر سطوح برخی عوامل خطرزای قلبی‌عروقی مردان سالمند با اضافه وزن بود. مواد و روش ها در این مطالعه نیمه‌تجربی، 24 مرد سالمند با اضافه وزن بر اساس نمایه توده بدن در دو گروه تجربی (12نفر) و کنترل (12 نفر) با دامنه سنی در گروه تمرین تناوبی هوازی 34/3±05/73 و گروه کنترل 44/3±33/71 به روش نمونه‌گیری در دسترس و هدفمند انتخاب شدند. آزمودنی‌ها به مدت هشت هفته، سه روز در هر هفته و هر جلسه 45 تا 60 دقیقه به انجام تمرینات تناوبی هوازی پرداختند. نمونه خونی پیش از شروع و پس از پایان مداخله تمرینی جمع‌آوری شد. با استفاده از نرم‌افزار SPSS ویرایش 16، مقایسه میانگین‌های درون‌گروهی و بین‌گروهی از روش آماری به ترتیب تی همبسته و تحلیل کوواریانس (MANOVA) محاسبه شد و نتایج در سطح معنی‌داری 05/0>P تجزیه‌وتحلیل شدند. یافته ها هشت هفته تمرینی تناوبی هوازی به کاهش معنی‌دار متغیرهای وزن، نمایه توده بدن و درصد چربی بدن منجر شد (05/0>P). همچنین سطوح اندوتلین-1 سرمی در پایان دوره کاهش معنی‌دار یافت (005/0P=). سطح نیتریک اکساید نیز در پایان دوره افزایش معنی‌دار یافت (05/0≥P).  نتیجه گیری این نتایج حاکی از آن است که هشت هفته تمرین تناوبی هوازی از طریق افزایش نیتریک اکساید و کاهش اندوتلین-1 می‌تواند در بهبود سلامت قلب و عروق و کاهش خطر بیماری آتروسکلروز مؤثر باشد. بنابراین پیشنهاد می‌شود از تمرینات تناوبی هوازی برای پیشگیری از آثار سوء ناشی از افزایش بروز بیماری آترواسکلروزیس استفاده شود.

Title: تأثیر اجرای الگوی توانمندسازی خانواده‌محور برخودکارآمدی سالمندان با فشار خون بالا

http://salmandj.uswr.ac.ir/article-1-1281-fa.html

اهداف بیماری‌های غیرواگیردار از علل مهم و عمده مرگ‌ومیر در سالمندان هستند. فشار خون بالا شایع‌ترین بیماری قلبی‌عروقی است که سالمندان با آن مواجه می‌شوند. یکی از مدل‌های ارتقای بهداشتی، الگوی توانمندسازی خانواده‌محور است. هدف از این مطالعه، تعیین تأثیر اجرای الگوی توانمندسازی خانواده‌محور (FCEM) بر میزان خودکارآمدی سالمندان با فشار خون بالا تحت پوشش شبکه بهداشت و درمان لنجان در سال 1393 بود. مواد و روش ها این پژوهش کارآزمایی بالینی روی 62 سالمندان مبتلا به فشار خون بالا در سال 1393 انجام شد. پس از نمونه‌گیری خوشه‌ای، نمونه‌ها به صورت تصادفی به دو گروه آزمون و کنترل تقسیم شدند. برای گروه آزمون، الگوی توانمندسازی خانواده‌محور و برای گروه کنترل مراقبت رایج و یک جلسه آموزشی اجرا شد. ابزارهای پژوهش چک‌لیست اطلاعات جمعیت‌شناختی و پرسش‌نامه توانمندسازی سنجش بعد خودکارآمدی بودند. پس‌آزمون یک هفته بعد انجام و یک‌ماه‌ونیم بعد پیگیری شد. از آزمون‌های آماری تی مستقل و آنالیز واریانس و نرم‌افزار Spss 20 برای تحلیل داده‌ها استفاده شد. یافته ها میانگین نمره بعد خودکارآمدی، قبل از مداخله در دو گروه آزمون و کنترل همسان بود (05/0P>)، اما یک هفته بعد از مداخله و یک‌ماه‌ونیم بعد از مداخله تفاوت معناداری در گروه آزمون پیدا شد (001/0P<).  نتیجه گیری در مطالعه حاضر اجرای الگوی توانمندسازی خانواده‌محور باعث ارتقای خودکارآمدی سالمندان مبتلا به فشار خون بالا شده است.

Title: پیش‌بینی بهزیستی روان‌شناختی سالمندان براساس توان مدیریت استرس و حمایت اجتماعی

http://salmandj.uswr.ac.ir/article-1-1299-fa.html

اهداف با توجه به اینکه پیوندهای بین‌فردی حمایت اجتماعی می‌تواند سلامتی را ارتقا دهد، در شرایط پراسترس حمایت اجتماعی نقش تعدیل‌کننده و محافظ را در مقابل آثار زیان‌آور فشار دارد. پژوهش حاضر با هدف پیش‌بینی رابطه بهزیستی روان‌شناختی با توان مدیریت استرس و حمایت اجتماعی انجام شد. مواد و روش ها این مطالعه توصیفی‌تحلیلی از نوع همبستگی و به شیوه مقطعی انجام شد. جامعه آماری شامل تمامی سالمندان ساکن شهر تهران (60ساله و بیشتر) می‌شد. نمونه مورد پژوهش به روش نمونه‌گیری در دسترس از سالمندان مراجعه‌کننده به پارک‌های منطقه 8 استان تهران در اردیبهشت و خرداد 1395 انتخاب شد. اندازه نمونه با استفاده از فرمول کوکران 131 نفر تعیین شد. در این پژوهش از فرم کوتاه مقیاس بهزیستی روان‌شناختی ریف (1989)، مقیاس مدیریت استرس کالزبیک (1990) و مقیاس حمایت اجتماعی فیلیپس (1977) استفاده شده است. تجزیه‌وتحلیل داده از طریق رگرسیون چندگانه انجام شد. یافته ها نتایج نشان می‌دهد حمایت اجتماعی با t=2/23 در سطح معناداری 05/0 و مدیریت استرس با  t=4/79در سطح  معناداری 01/0، بهزیستی روان‌شناختی را پیش‌بینی می‌کند.  نتیجه گیری بر اساس یافته‌های این مطالعه، متغیرهای حمایت اجتماعی و مدیریت استرس می‌توانند متغیر بهزیستی روان‌شناختی در سالمندان را پیش‌بینی کنند. بنابراین مداخلات کمک‌کننده برای سالمندان به منظور کسب و نگهداری منابع مؤثر و مهارت‌های مقابله، برای ترویج بیشتر روند سالمندی موفق و به همین ترتیب استفاده از سبک‌های مقابله فعال توصیه می‌شود.

Title: بررسی تأثیر آموزش مؤلفه‌های هوش معنوی بر میزان اضطراب مرگ در سالمندان

http://salmandj.uswr.ac.ir/article-1-1346-fa.html

اهداف این پژوهش با هدف بررسی تأثیر آموزش مؤلفه‌های هوش معنوی بر میزان اضطراب مرگ در سالمندان مقیم سرای سالمندان شهر سنندج انجام شد.  مواد و روش ها روش پژوهش از لحاظ هدف کاربردی و از لحاظ روش اجرا نیمه آزمایشی از نوع پیش‌آزمون پس‌آزمون با گروه کنترل بود. جامعه آماری پژوهش را تمامی افراد ساکن سرای سالمندان در شهر سنندج در نیمه اول سال 1395 تشکیل داده‌اند. از این جامعه آماری و از بین کسانی که در مقیاس اضطراب مرگ نمره بالایی دریافت کرده بودند، 20 نفر به شیوه در دسترس انتخاب و در دو گروه 10 نفری (گروه آزمایش و گروه کنترل) جایگزین شدند. مقیاس اضطراب مرگ کالت-لستر (1969) به عنوان پیش‌آزمون روی آزمودنی‌ها اجرا شد. سپس گروه آزمایشی مداخله مبتنی بر رویکرد آموزش، مفاهیم هوش معنوی را در هشت جلسه به مدت 90 دقیقه دریافت کردند. طی این مدت گروه کنترل هیچ‌گونه مداخله‌ای را دریافت نکردند. بعد از اتمام جلسات مداخله، پس‌آزمون روی آزمودنی‌ها اجرا شد. داده‌ها با استفاده از تحلیل کواریانس یک راهه و چندراهه و ازطریق نرم افزار spss-22 تحلیل شدند.  یافته ها میانگین و انحراف معیار سنی گروه آزمایش 68/5±5/62 و گروه کنترل 33/4±1/63 سال بود. نتایج تحلیل کوواریانس تفاوت معنا‌داری در مرحله پس‌آزمون بین گروه آزمایش (81/4±9/120) و گروه کنترل (65/8±3/131) نشان داد (01/0P≤). بنابراین مشخص شد آموزش مؤلفه‌های هوش معنوی باعث کاهش اضطراب مرگ سالمندان شده است. همچنین آموزش مؤلفه‌های هوش معنوی باعث کاهش دو بعد اضطراب (مرگ اطرافیان و دیدن اطرافیانتان درحال‌مرگ) در سالمندان شد (01/0P≤). اما در کاهش ابعاد اضطراب مرگ در خصوص مرگ خود و دیدن خود درحال‌مرگ نتایج معنا‌داری مشاهده نشد.  نتیجه گیری بر اساس یافته‌های پژوهش، آموزش مؤلفه‌های هوش معنوی در کاهش اضطراب مرگ مؤثر است. بنابراین توصیه می‌شود در مراکز سالمندان به آموزش جنبه‌های معنوی به سالمندان توجه بیشتری مبذول شود.
